# 4-(Dimethylamino)pyridinium dibromidotriphenyl­stannate(IV)

**DOI:** 10.1107/S1600536808011094

**Published:** 2008-04-26

**Authors:** Ilyana Norhafiza, Kong Mun Lo, Seik Weng Ng

**Affiliations:** a Department of Chemistry, University of Malaya, 50603 Kuala Lumpur, Malaysia

## Abstract

The anion in the title salt, (C_7_H_11_N_2_)[SnBr_2_(C_6_H_5_)_3_], lies on a twofold rotation axis that passes through the metal atom as well as the C_*ipso*_—C_*para*_ atoms of one of the aromatic rings. The metal center is five-coordinate in a *trans*-Br_2_SnC_3_ trigonal bipyramidal geometry. The cation is disordered about a center of inversion.

## Related literature

For the crystal structures of dibromidotriorganostannates, see: Aslanov *et al.* (1977 ▶); Spek *et al.* (2004 ▶); Wharf & Simard (1991 ▶).
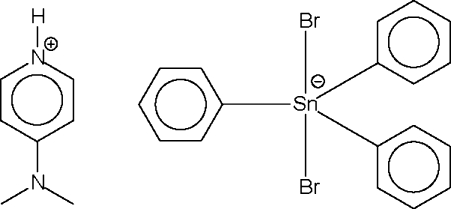


## Experimental

### 

#### Crystal data

(C_7_H_11_N_2_)[SnBr_2_(C_6_H_5_)_3_]
*M* *_r_* = 632.99
Monoclinic, 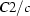

*a* = 15.5955 (2) Å
*b* = 10.6897 (1) Å
*c* = 14.8204 (2) Å
β = 93.924 (1)°
*V* = 2464.93 (5) Å^3^
*Z* = 4
Mo *K*α radiation
μ = 4.29 mm^−1^
*T* = 100 K
0.3 × 0.2 × 0.1 mm

#### Data collection

Bruker SMART APEX diffractometer
Absorption correction: multi-scan (*SADABS*; Sheldrick, 1996 ▶) *T*_min_ = 0.368, *T*_max_ = 0.651
24740 measured reflections
2840 independent reflections
2328 reflections with *I* > 2σ(*I*)
*R*_int_ = 0.033

#### Refinement

*R*[*F* ^2^ > 2σ(*F* ^2^)] = 0.026
*wR*(*F* ^2^) = 0.069
*S* = 1.05
2840 reflections
180 parameters
69 restraints
H-atom parameters constrained
Δρ_max_ = 0.34 e Å^−3^
Δρ_min_ = −1.16 e Å^−3^

### 

Data collection: *APEX2* (Bruker, 2007 ▶); cell refinement: *SAINT* (Bruker, 2007 ▶); data reduction: *SAINT*; program(s) used to solve structure: *SHELXS97* (Sheldrick, 2008 ▶); program(s) used to refine structure: *SHELXL97* (Sheldrick, 2008 ▶); molecular graphics: *X-SEED* (Barbour, 2001 ▶); software used to prepare material for publication: *publCIF* (Westrip, 2008 ▶).

## Supplementary Material

Crystal structure: contains datablocks global, I. DOI: 10.1107/S1600536808011094/sg2227sup1.cif

Structure factors: contains datablocks I. DOI: 10.1107/S1600536808011094/sg2227Isup2.hkl

Additional supplementary materials:  crystallographic information; 3D view; checkCIF report

## Figures and Tables

**Table:** 

Sn1—C1	2.135 (2)
Sn1—C7	2.149 (3)
Sn1—Br1	2.7801 (3)

**Table:** 

C1—Sn1—C1^i^	116.17 (13)
C1—Sn1—C7	121.92 (6)
C1—Sn1—Br1	88.61 (6)
C1—Sn1—Br1^i^	90.68 (6)
C7—Sn1—Br1	90.68 (1)
Br1—Sn1—Br1^i^	178.64 (2)

Symmetry code: (i) 
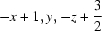
.

**Fig. 1.**
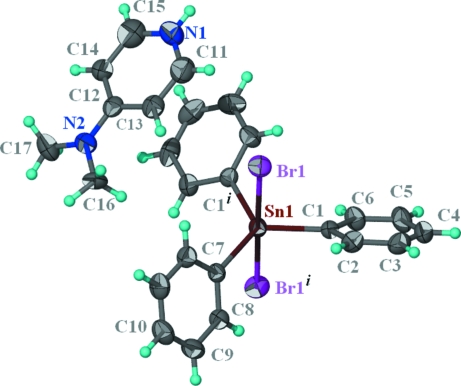
Thermal ellipsoid plot of the title compound.

## supplementary crystallographic information

### Comment

The aqueous solubility of biocidal triorganotin halides can be improved by converting them to their ionic salts through treatment with an ammonium halide. A small number of ammonium dihalogenotriorganostannates are known; tetraethylammonium dibromidotriphenylstannate, who crystal structure is known, is synthesized in this manner (Wharf & Simard, 1991).

The present synthesis uses the mild brominating agent, 4-dimethylaminopyridine hydrobromide perbromide, which cleaves one of the four tin-carbon bonds of tetraphenyltin to yield the bromidotriphenylstannate anion. In the title compound (I), the anion lies about a twofold rotation axis that passes through the metal atom as well as the C*_ipso_*–C*_para_* atoms of one of the aromatic rings. The metal center is five-coordinate in a *trans*-C_3_SnBr_2_ trigonal bipyramidal geometry. The cation and anion exist as two non-interacting species (Fig. 1).

### Experimental

Tetraphenyltin (2 g, 4.7 mmol) and 4-dimethylaminopyridine hydrobromide perbromide (1.7 g, 4.6 mmol) were heated in chloroform (100 ml) for 6 h. The solution was filtered and the solvent allow to evaporate to give yellow crystals (m.p. 455–457 K, 80% yield).

### Refinement

The cation is disordered over a center-of-inversion, and was allowed to refine over this symmetry element as a half-occupancy cation. For the aromatic ring, 1,2-related distances were restrained to 1.39±0.01 Å and 1,3-related ones to 2.78±0.01 Å. For the dimethylamino group, the N–C distances were restrained to 1.50±0.01 Å. Anisotropic temperature factors of the carbon and nitrogen atoms were restrained to be nearly isotropic.

Carbon-bound H-atoms were placed in calculated positions (C—H 0.93 to 0.97 Å) and were included in the refinement in the riding model approximation, with *U*(H) set to 1.2 to 1.5*U*(C). The ammonium H-atom was similarly treated (N–H 0.86 Å; *U*(H) = 1.2*U*(B).

The final difference Fourier map had a large peak at 1 Å from Br1.

### Crystal data

**Table:**

(C_7_H_11_N_2_)[SnBr_2_(C_6_H_5_)_3_]	*F*_000_ = 1240
*M**_r_* = 632.99	*D*_x_ = 1.706 Mg m^−^^3^
Monoclinic, *C*2/*c*	Mo *K*α radiation λ = 0.71073 Å
Hall symbol: -C 2yc	Cell parameters from 7191 reflections
*a* = 15.5955 (2) Å	θ = 2.3–24.8º
*b* = 10.6897 (1) Å	µ = 4.29 mm^−^^1^
*c* = 14.8204 (2) Å	*T* = 100 K
β = 93.924 (1)º	Block, yellow
*V* = 2464.93 (5) Å^3^	0.3 × 0.2 × 0.1 mm
*Z* = 4	

### Data collection

**Table:**

Bruker SMART APEXII diffractometer	2840 independent reflections
Radiation source: fine-focus sealed tube	2328 reflections with *I* > 2σ(*I*)
Monochromator: graphite	*R*_int_ = 0.033
*T* = 100 K	θ_max_ = 27.5º
φ and ω scans	θ_min_ = 2.3º
Absorption correction: multi-scan(SADABS; Sheldrick, 1996)	*h* = −20→20
*T*_min_ = 0.368, *T*_max_ = 0.651	*k* = −13→13
24740 measured reflections	*l* = −19→19

### Refinement

**Table:**

Refinement on *F*^2^	Secondary atom site location: difference Fourier map
Least-squares matrix: full	Hydrogen site location: inferred from neighbouring sites
*R*[*F*^2^ > 2σ(*F*^2^)] = 0.026	H-atom parameters constrained
*wR*(*F*^2^) = 0.069	*w* = 1/[σ^2^(*F*_o_^2^) + (0.0352*P*)^2^ + 2.1489*P*] where *P* = (*F*_o_^2^ + 2*F*_c_^2^)/3
*S* = 1.06	(Δ/σ)_max_ = 0.001
2840 reflections	Δρ_max_ = 0.34 e Å^−^^3^
180 parameters	Δρ_min_ = −1.16 e Å^−^^3^
69 restraints	Extinction correction: none
Primary atom site location: structure-invariant direct methods	

### Fractional atomic coordinates and isotropic or equivalent isotropic displacement parameters (Å^2^)

**Table:**

	*x*	*y*	*z*	*U*_iso_*/*U*_eq_	Occ. (<1)
Sn1	0.5000	0.76516 (2)	0.7500	0.03975 (9)	
Br1	0.56766 (2)	0.76825 (3)	0.928473 (19)	0.05538 (10)	
C1	0.60873 (15)	0.8708 (2)	0.71394 (16)	0.0377 (5)	
C2	0.69169 (17)	0.8273 (3)	0.73324 (19)	0.0504 (6)	
H2	0.7004	0.7512	0.7629	0.061*	
C3	0.76163 (18)	0.8960 (3)	0.7087 (2)	0.0613 (8)	
H3	0.8169	0.8647	0.7206	0.074*	
C4	0.7501 (2)	1.0094 (3)	0.6672 (2)	0.0647 (9)	
H4	0.7974	1.0557	0.6515	0.078*	
C5	0.6686 (2)	1.0548 (3)	0.6489 (2)	0.0648 (8)	
H5	0.6603	1.1322	0.6210	0.078*	
C6	0.59864 (18)	0.9851 (3)	0.6720 (2)	0.0518 (7)	
H6	0.5435	1.0162	0.6588	0.062*	
C7	0.5000	0.5641 (3)	0.7500	0.0372 (7)	
C8	0.51067 (16)	0.4971 (3)	0.67160 (19)	0.0461 (6)	
H8	0.5173	0.5399	0.6179	0.055*	
C9	0.51168 (18)	0.3672 (3)	0.6717 (2)	0.0557 (7)	
H9	0.5203	0.3239	0.6187	0.067*	
C10	0.5000	0.3029 (4)	0.7500	0.0595 (11)	
H10	0.5000	0.2159	0.7500	0.071*	
N1	0.3033 (4)	0.9766 (5)	1.0723 (4)	0.0750 (16)	0.50
H1	0.3207	1.0468	1.0950	0.090*	0.50
C11	0.3619 (5)	0.8914 (9)	1.0461 (4)	0.065 (4)	0.50
H11	0.4203	0.9101	1.0530	0.078*	0.50
C12	0.3361 (4)	0.7792 (6)	1.0100 (4)	0.0548 (14)	0.50
H12	0.3757	0.7209	0.9920	0.066*	0.50
C13	0.2490 (6)	0.7552 (8)	1.0011 (6)	0.0454 (9)	0.50
C14	0.1886 (3)	0.8397 (5)	1.0268 (4)	0.0547 (14)	0.50
H14	0.1302	0.8215	1.0199	0.066*	0.50
C15	0.2171 (6)	0.9515 (9)	1.0628 (6)	0.091 (9)	0.50
H15	0.1778	1.0104	1.0808	0.109*	0.50
N2	0.2210 (3)	0.6420 (4)	0.9598 (3)	0.0512 (11)	0.50
C16	0.2813 (5)	0.5487 (7)	0.9338 (7)	0.059 (6)	0.50
H16A	0.3126	0.5803	0.8850	0.089*	0.50
H16B	0.2508	0.4744	0.9145	0.089*	0.50
H16C	0.3208	0.5295	0.9844	0.089*	0.50
C17	0.1314 (6)	0.6173 (12)	0.9365 (9)	0.077 (4)	0.50
H17A	0.0998	0.6239	0.9897	0.116*	0.50
H17B	0.1250	0.5345	0.9119	0.116*	0.50
H17C	0.1096	0.6772	0.8924	0.116*	0.50

### Atomic displacement parameters (Å^2^)

**Table:**

	*U*^11^	*U*^22^	*U*^33^	*U*^12^	*U*^13^	*U*^23^
Sn1	0.03660 (13)	0.03341 (14)	0.04972 (15)	0.000	0.00641 (10)	0.000
Br1	0.05950 (19)	0.0641 (2)	0.04166 (16)	−0.00651 (13)	−0.00302 (13)	−0.00418 (12)
C1	0.0366 (12)	0.0376 (13)	0.0393 (12)	−0.0018 (10)	0.0044 (10)	−0.0026 (10)
C2	0.0446 (14)	0.0523 (17)	0.0544 (16)	0.0075 (12)	0.0036 (12)	0.0014 (13)
C3	0.0356 (14)	0.085 (2)	0.0640 (19)	−0.0006 (14)	0.0056 (13)	−0.0193 (17)
C4	0.0598 (19)	0.073 (2)	0.0630 (19)	−0.0290 (17)	0.0163 (15)	−0.0189 (17)
C5	0.078 (2)	0.0459 (17)	0.070 (2)	−0.0193 (15)	0.0078 (17)	0.0074 (15)
C6	0.0485 (15)	0.0409 (15)	0.0651 (18)	−0.0002 (12)	−0.0021 (13)	0.0052 (13)
C7	0.0311 (16)	0.0348 (17)	0.0456 (19)	0.000	0.0023 (14)	0.000
C8	0.0429 (14)	0.0454 (14)	0.0503 (15)	−0.0012 (11)	0.0056 (11)	−0.0031 (12)
C9	0.0529 (16)	0.0472 (16)	0.0668 (19)	0.0020 (13)	0.0029 (14)	−0.0157 (14)
C10	0.055 (2)	0.038 (2)	0.085 (3)	0.000	−0.001 (2)	0.000
N1	0.080 (4)	0.075 (4)	0.070 (3)	−0.024 (3)	0.002 (3)	−0.017 (3)
C11	0.057 (5)	0.077 (7)	0.061 (5)	−0.010 (5)	−0.003 (4)	−0.002 (4)
C12	0.050 (3)	0.066 (4)	0.049 (3)	0.000 (3)	0.003 (2)	0.004 (3)
C13	0.051 (2)	0.050 (2)	0.0354 (18)	−0.0006 (18)	0.0071 (16)	0.0045 (17)
C14	0.050 (3)	0.058 (3)	0.057 (3)	−0.004 (3)	0.009 (3)	−0.007 (3)
C15	0.096 (12)	0.091 (12)	0.088 (12)	−0.004 (8)	0.017 (8)	−0.006 (8)
N2	0.061 (3)	0.046 (2)	0.047 (3)	−0.002 (2)	0.003 (2)	−0.001 (2)
C16	0.068 (8)	0.049 (7)	0.061 (8)	0.005 (5)	0.014 (5)	−0.018 (5)
C17	0.073 (7)	0.068 (6)	0.088 (6)	−0.018 (5)	−0.005 (5)	−0.006 (5)

### Geometric parameters (Å, °)

**Table:**

Sn1—C1	2.135 (2)	C10—C9^i^	1.372 (4)
Sn1—C1^i^	2.135 (2)	C10—H10	0.9300
Sn1—C7	2.149 (3)	N1—C11	1.366 (9)
Sn1—Br1^i^	2.7801 (3)	N1—C15	1.369 (9)
Sn1—Br1	2.7801 (3)	N1—H1	0.8600
C1—C6	1.375 (4)	C11—C12	1.363 (9)
C1—C2	1.386 (3)	C11—H11	0.9300
C2—C3	1.384 (4)	C12—C13	1.379 (9)
C2—H2	0.9300	C12—H12	0.9300
C3—C4	1.365 (5)	C13—C14	1.378 (9)
C3—H3	0.9300	C13—N2	1.412 (8)
C4—C5	1.371 (5)	C14—C15	1.370 (9)
C4—H4	0.9300	C14—H14	0.9300
C5—C6	1.383 (4)	C15—H15	0.9300
C5—H5	0.9300	N2—C16	1.441 (7)
C6—H6	0.9300	N2—C17	1.441 (8)
C7—C8	1.384 (3)	C16—H16A	0.9600
C7—C8^i^	1.384 (3)	C16—H16B	0.9600
C8—C9	1.389 (4)	C16—H16C	0.9600
C8—H8	0.9300	C17—H17A	0.9600
C9—C10	1.372 (4)	C17—H17B	0.9600
C9—H9	0.9300	C17—H17C	0.9600
			
C1—Sn1—C1^i^	116.17 (13)	C9—C8—C7	121.2 (3)
C1—Sn1—C7	121.92 (6)	C9—C8—H8	119.4
C1^i^—Sn1—C7	121.92 (6)	C7—C8—H8	119.4
C1—Sn1—Br1	88.61 (6)	C10—C9—C8	120.0 (3)
C1—Sn1—Br1^i^	90.68 (6)	C10—C9—H9	120.0
C1^i^—Sn1—Br1^i^	88.61 (6)	C8—C9—H9	120.0
C7—Sn1—Br1	90.68 (1)	C9—C10—C9^i^	119.8 (4)
C7—Sn1—Br1^i^	90.68 (1)	C9—C10—H10	120.1
C1^i^—Sn1—Br1	90.68 (6)	C9^i^—C10—H10	120.1
Br1—Sn1—Br1^i^	178.64 (2)	C11—N1—C15	120.8 (7)
C6—C1—C2	117.9 (2)	C11—N1—H1	119.6
C6—C1—Sn1	120.98 (18)	C15—N1—H1	119.6
C2—C1—Sn1	121.13 (19)	C12—C11—N1	120.8 (7)
C3—C2—C1	120.6 (3)	C12—C11—H11	119.6
C3—C2—H2	119.7	N1—C11—H11	119.6
C1—C2—H2	119.7	C11—C12—C13	117.7 (6)
C4—C3—C2	120.5 (3)	C11—C12—H12	121.2
C4—C3—H3	119.7	C13—C12—H12	121.2
C2—C3—H3	119.7	C14—C13—C12	122.6 (7)
C3—C4—C5	119.7 (3)	C14—C13—N2	119.0 (7)
C3—C4—H4	120.2	C12—C13—N2	118.3 (7)
C5—C4—H4	120.2	C15—C14—C13	118.0 (7)
C4—C5—C6	119.8 (3)	C15—C14—H14	121.0
C4—C5—H5	120.1	C13—C14—H14	121.0
C6—C5—H5	120.1	C14—C15—N1	120.1 (7)
C1—C6—C5	121.5 (3)	C14—C15—H15	120.0
C1—C6—H6	119.2	N1—C15—H15	120.0
C5—C6—H6	119.2	C13—N2—C16	121.4 (6)
C8—C7—C8^i^	117.7 (3)	C13—N2—C17	121.8 (7)
C8—C7—Sn1	121.13 (17)	C16—N2—C17	116.7 (7)
C8^i^—C7—Sn1	121.13 (17)		
			
C1^i^—Sn1—C1—C6	−32.12 (19)	C1—Sn1—C7—C8^i^	119.08 (14)
C7—Sn1—C1—C6	147.88 (19)	C1^i^—Sn1—C7—C8^i^	−60.92 (14)
Br1^i^—Sn1—C1—C6	56.7 (2)	Br1^i^—Sn1—C7—C8^i^	−149.70 (12)
Br1—Sn1—C1—C6	−122.2 (2)	Br1—Sn1—C7—C8^i^	30.30 (12)
C1^i^—Sn1—C1—C2	146.9 (2)	C8^i^—C7—C8—C9	−0.75 (19)
C7—Sn1—C1—C2	−33.1 (2)	Sn1—C7—C8—C9	179.25 (19)
Br1^i^—Sn1—C1—C2	−124.3 (2)	C7—C8—C9—C10	1.5 (4)
Br1—Sn1—C1—C2	56.9 (2)	C8—C9—C10—C9^i^	−0.74 (18)
C6—C1—C2—C3	−1.6 (4)	C15—N1—C11—C12	−0.1 (3)
Sn1—C1—C2—C3	179.4 (2)	N1—C11—C12—C13	−0.1 (3)
C1—C2—C3—C4	1.8 (5)	C11—C12—C13—C14	0.2 (7)
C2—C3—C4—C5	−0.7 (5)	C11—C12—C13—N2	177.0 (7)
C3—C4—C5—C6	−0.4 (5)	C12—C13—C14—C15	−0.2 (10)
C2—C1—C6—C5	0.4 (4)	N2—C13—C14—C15	−177.0 (7)
Sn1—C1—C6—C5	179.5 (2)	C13—C14—C15—N1	0.1 (9)
C4—C5—C6—C1	0.6 (5)	C11—N1—C15—C14	0.1 (7)
C1—Sn1—C7—C8	−60.92 (14)	C14—C13—N2—C16	−178.4 (7)
C1^i^—Sn1—C7—C8	119.08 (14)	C12—C13—N2—C16	4.7 (11)
Br1^i^—Sn1—C7—C8	30.30 (12)	C14—C13—N2—C17	5.7 (12)
Br1—Sn1—C7—C8	−149.70 (12)	C12—C13—N2—C17	−171.2 (9)

Symmetry codes: (i) −*x*+1, *y*, −*z*+3/2.
